# Secondhand smoke exposure induces Raf/ERK/MAPK-mediated upregulation of cerebrovascular endothelin ET_A _receptors

**DOI:** 10.1186/1471-2202-12-109

**Published:** 2011-11-01

**Authors:** Lei Cao, Cang-Bao Xu, Yaping Zhang, Yong-Xiao Cao, Lars Edvinsson

**Affiliations:** 1 Division of Experimental Vascular Research, Institute of Clinical Science in Lund, Lund University, Sweden; 2 Department of Pharmacology, Xi'an Jiaotong University College of Medicine, Xi'an, Shaanxi, P. R. China

## Abstract

**Background:**

Cigarette smoking enhances the risk of stroke. However, the underlying molecular mechanisms are largely unknown. The present study established an *in vivo *rat secondhand cigarette smoking (SHS) model and examined the hypothesis that SHS upregulates endothelin receptors with increased cerebrovascular contraction *via *the Raf/extracellular signal-regulated kinase (ERK)/mitogen-activated protein kinases (MAPK) pathway.

**Results:**

Rats were exposed to SHS for up to 8 weeks. The cerebral artery vasoconstriction was recorded by a sensitive myograph. The mRNA and protein expressions for endothelin receptors in cerebral arteries were studied by real-time PCR and Western blot. Compared to fresh air exposed rats, cerebral arteries from SHS rats exhibited stronger contractile responses (*P *< 0.05) mediated by endothelin type A (ET_A_) receptors. The expressions of mRNA and protein for ET_A _receptors in the cerebral arteries from SHS rats were higher (*P *< 0.05) than that in control. SHS did not affect endothelin type B (ET_B_) receptor-mediated contractions, mRNA or protein levels. The results suggest that SHS upregulates ET_A_, but not ET_B _receptors *in vivo*. After SHS exposure, the mRNA levels of Raf-1 and ERK1/2, the protein expression of phosphorylated (p)-Raf-1 and p-ERK1/2 were increased (*P *< 0.05). Raf-1 inhibitor, GW5074 suppressed the enhanced ET_A _receptor-mediated contraction, mRNA and protein levels induced by SHS. In addition, GW5074 inhibited the SHS-caused increased mRNA and phosphorylated protein levels of Raf-1 and ERK1/2, suggesting that SHS induces activation of the Raf/ERK/MAPK pathway.

**Conclusions:**

SHS upregulates cerebrovascular ET_A _receptors *via *the Raf/ERK/MAPK pathway, which provides novel understanding of mechanisms involved in SHS-associated stroke.

## Background

Passive smoke exposure or secondhand smoke (SHS) is strongly associated with ischemic and hemorrhagic stroke [1], and has harmful effects on the structure and function of cerebral blood vessels, promoting atherosclerosis and stiffening of arteries [2]. However, the biological basis of SHS on the vessel walls is not well understood.

Endothelin (ET)-1 is one of the most potent vasoconstriction found in the circulation with elevated levels in stroke [3]. ET-1 is produced by endothelial cells, mediates its vasomotor response through two different G-protein coupled receptors, the endothelin type A (ET_A_) and the endothelin type B (ET_B_) receptor [4]. In cerebral vessels, the ET_A _receptors are found mainly on the smooth muscle cells and mediate strong vasoconstriction [3], while ET_B _receptors are primarily situated on the endothelium of cerebral vessels and stimulate the formation of nitric oxide and prostacyclin mediating vasodilatation [5]. Because ET-1 causes potent and long-lasting vasoconstriction, and there are increased levels of ET-1 in cerebral spinal fluid (CSF) after subarachnoid hemorrhage (SAH) [6,7]; it has been suggested to play an important role in the pathogenesis of delayed cerebral vasospasm following SAH [8] and in cerebral ischemia [9]. In addition, there are increasing evidences demonstrating that experimental SAH and cerebral ischemia may be associated with ET receptor upregulation in cerebral artery smooth muscle cells [10,11].

The main risk factors for stroke in general include hyperlipidemia, hypertension and cigarette smoking [12]. Here we address in particular one of these, SHS, which is associated with increased risk of SAH and ischemic stroke in general population [13]. Our previous *in vitro *studies have demonstrated that lipid-soluble smoke particles, but not water soluble smoke particles or nicotine *per se*, induce ET_B _receptor upregulation in cerebral vessels [14]. The increased receptors result in enhanced contractility and local inflammation. To the best of our knowledge, it has not been studied if SHS *in vivo *is associated with elevated expression of ET receptors. If both the formation of ET-1 and the number of contractile ET receptors are increased in individuals after exposure to SHS, it may bring about larger damage in SAH or cerebral infarct, compared to the non-smokers. We hypothesize that SHS exposure *in vivo *upregulates ET receptors in cerebral arteries, which may in turn contribute to larger brain damage in stroke among smoke exposed subjects.

The cellular mechanisms involved in SHS-associated stroke are unclear; here we examine if the ET receptor upregulation induced by SHS is associated with intracellular mitogen-activated protein kinase (MAPK) signaling. This system consists of extracellular signal-regulated protein kinase 1 and 2 (ERK1/2), c-Jun N-terminal kinase (JNK) and p38 pathways. Raf-1 is the initial protein kinase in the MAPK signal transduction pathway which phosphorylates subsequent MAP kinase/extracellular signal-regulated kinase kinase 1 and 2 (MEK1/2) [15]. We have recently in detail described that activation of MAPK-mediated signal transduction is associated with upregulation of ET receptors in cerebral vasculature and that ET receptor expression is enhanced in ischemic stroke [3]. The importance of MAPK signaling in the pathophysiology of ischemic stroke has been widely studied. Increased ERK1/2 phosphorylation has been observed in the ischemic area after both transient and permanent middle cerebral occlusion, as well as after global ischemia [16,17]. Consequently, inhibitors of ERK1/2 and MEK1/2 have been effective in reducing the infarct size in cerebral ischemia [18,19], and in SAH [20]. ERK1/2 is also activated in the cerebral arteries of the ischemic brain, pointing towards a role in vascular alterations [21]. However, it is not known if the risk factor SHS *per se *may alter ET receptor expression in cerebral arteries and if this is associated with intracellular signaling *via *the Raf/ERK/MAPK pathway.

The present study was designed, using an *in vivo *rat passive smoke exposure model, to demonstrate that cigarette smoke may upregulate cerebrovascular ET receptors, and to examine the intracellular signal mechanisms of SHS-induced enhanced ET receptor expression by *in vivo *treatment with a specific Raf-1 inhibitor.

## Results

### General

There was no significant difference in cerebral artery contractile responses to K^+^, sarafotoxin 6 c (S6c) and ET-1 after 2 or 4 weeks in SHS exposed rats as compared to rats exposed to fresh air for a similar time period (Table 1). Therefore, we only present detailed results from the 8 weeks of exposure to SHS.

**Table 1:** Time course of SHS on rat cerebral artery contractions induced by K^+^, S6c and ET-1.

Exposure time (week)	Group	*n*	K^+ ^(mN)	S6c (E_max _% of K^+^)	ET-1 (E_max _% of K^+^)
2	Fresh air	4	5.6 ± 0.58	8.5 ± 0.9	127 ± 14
	SHS	3	5.8 ± 0.63	8.3 ± 1.0	132 ± 15
4	Fresh air	4	5.4 ± 0.52	8.9 ± 1.1	131 ± 17
	SHS	4	6.2 ± 0.69	9.1 ± 1.3	136 ± 19

The cerebral arteries were from groups of fresh air and SHS for 2 and 4 weeks. K^+^-induced responses were expressed as absolute values of contraction (mN). S6c and ET-1-induced responses were expressed as percentage of K^+^-induced contraction. E_max_, maximal contraction; SHS, secondhand smoke exposure.

### Effects of SHS on ET receptor-mediated contractions in cerebral artery

The contraction elicited by K^+ ^was used as a reference for the contractile capacity. K^+^- induced contractile responses did not differ significantly in artery segments from fresh air, SHS and SHS plus inhibitor groups (Table 2). The ET_B _receptor-mediated contraction was examined using the specific ET_B _receptor agonist S6c, which has been characterized in detail before using the ET_B _receptor antagonist IRL2500 [22]. The vasoconstriction induced by a combined ET_A _and ET_B _receptor agonist ET-1 was studied after desensitizing the ET_B _receptors with S6c prior to adding ET-1, leaving only ET_A _receptors to respond. This has been verified by use of the selective ET_A _receptor antagonist FR139317 [22,23].

**Table 2:** Effect of 8 week SHS on rat cerebral artery contractions induced by K^+^, S6c and ET-1.

Group	*n*	K^+ ^(mN)	S6c		ET-1	
			
			E_max _(% of K^+^)	pEC_50_	E_max _(% of K^+^)	pEC_50_
Fresh air	10	5.7 ± 0.42	8.7 ± 0.5	9.32 ± 0.13	124 ± 9	8.86 ± 0.15
SHS	9	6.1 ± 0.53	9.5 ± 0.6	9.54 ± 0.19	160 ± 11*	8.97 ± 0.11
SHS+GW	8	6.3 ± 0.58	9.7 ± 0.9	9.37 ± 0.14	132 ± 15	8.77 ± 0.13

The cerebral arteries were from groups of fresh air, SHS and SHS treatment with GW5074 for 8 weeks. K^+^-induced responses were expressed as absolute values of contraction (mN). S6c and ET-1-induced responses were expressed as percentage of K^+^-induced contraction. *P < 0.05 versus fresh air group. E_max_, maximal contraction; pEC_50_, negative logarithm of the concentration that produces 50% of the maximal contractile effect; SHS, secondhand smoke exposure; GW, GW5074 (0.5 mg/kg).

**Figure 1:**
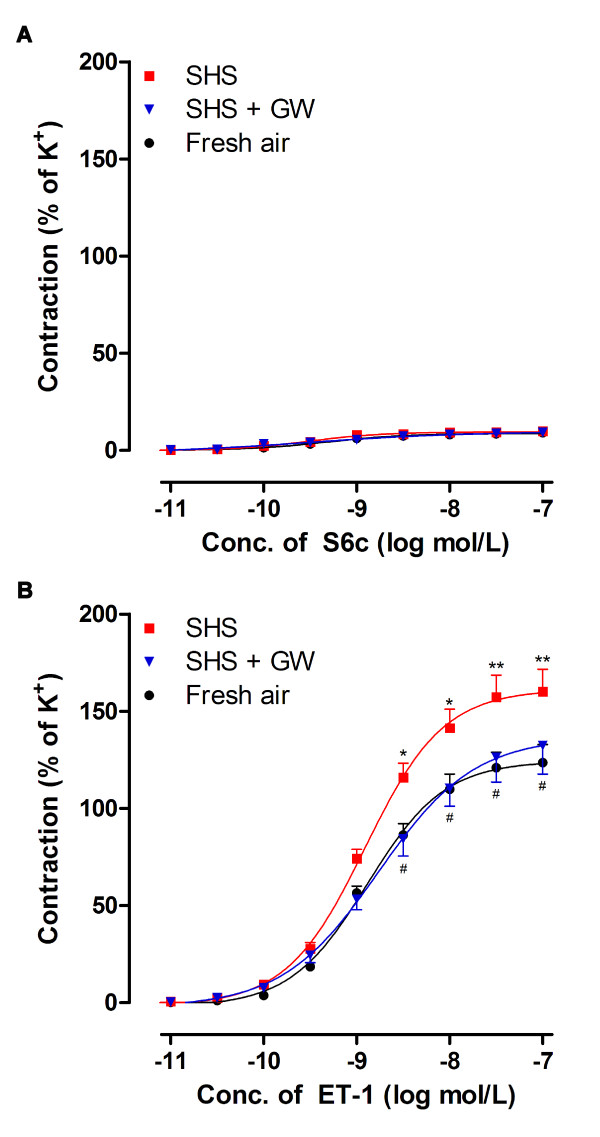
**Contractile responses induced by S6c (A) and ET-1 (B) in rat cerebral arteries**. The cerebral arteries were isolated from groups of fresh air, SHS and SHS treatment with GW5074. Contractions were induced by cumulative application of S6c or ET-1 (*n *= 8-10). *P < 0.05, **P < 0.01 versus fresh air group, ^#^*P *< 0.05 versus SHS group; SHS, secondhand smoke exposure; GW, GW5074 (0.5 mg/kg).

S6c only induced a slight contraction (< 10% of K^+^-induced contraction) in cerebral arteries of fresh air exposed rats. There was no significant difference in S6c-induced contractions between SHS and fresh air groups (Figure 1A). After desensitization of ET_B _receptors, cumulative administration of ET-1 induced potent contraction of fresh arteries in a concentration-dependent manner (Figure 1B) with an E_max _of 124 ± 9%. Following SHS the concentration-response curve showed an increased E_max _(160 ± 11%; *P *< 0.05) with no significant difference in pEC_50 _values (Table 2). This indicates that the efficacy of the response is increased after SHS exposure.

### Effects of SHS on ET receptor mRNA and protein expressions

The mRNA and protein levels of ET_B _and ET_A _receptors in cerebral arteries were measured by real-time PCR and Western blot, respectively. The standard curves of each primer pair in the qPCR had almost similar slopes, indicating that GAPDH and receptor cDNAs were amplified with the same efficiency (data not shown) [10]. The values of each slope were close to 3.3, meaning that the amplification efficiencies were almost optimal. There was no significant contaminating nucleic acid in blank control samples.

**Figure 2:**
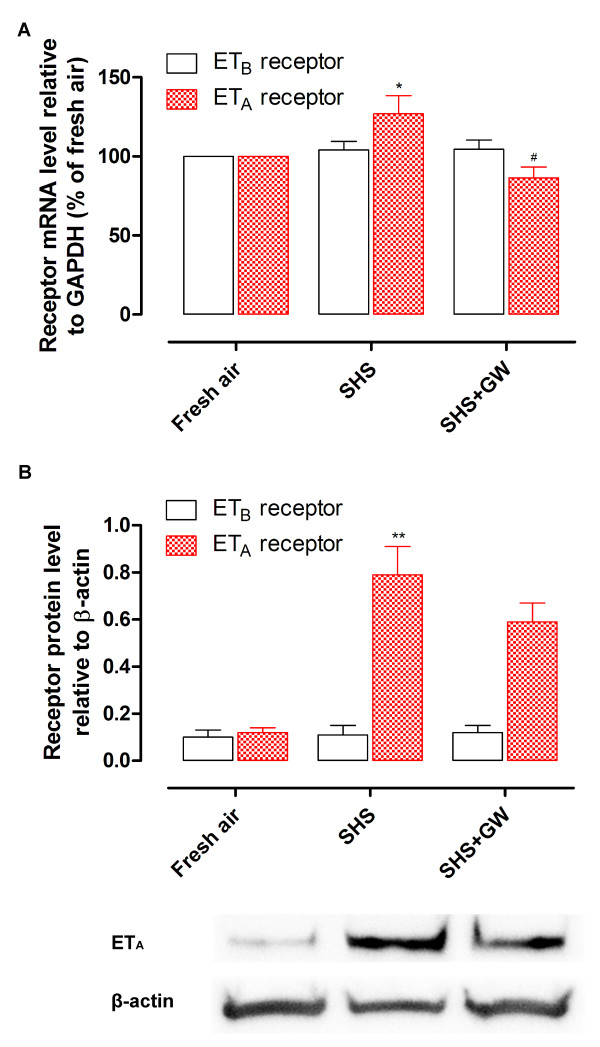
**The levels of ET_B _and ET_A _receptor mRNA (A) and protein (B) expression in rat cerebral arteries**. The cerebral arteries were isolated from groups of fresh air, SHS and SHS treatment with GW5074. Receptor mRNA content was examined by real-time PCR (*n *= 5-8) and the protein level was examined by Western blot (*n *= 5-6). *P < 0.05, **P < 0.01 versus fresh air group, ^#^*P *< 0.05 versus SHS group; SHS, secondhand smoke exposure; GW, GW5074 (0.5 mg/kg).

The ET_B _receptor mRNA expression remained unaltered after SHS exposure as compared to control (Figure 2A). The protein level of ET_B _receptor relative to β-actin was 0.10 ± 0.03 in fresh air exposed rats, and 0.11 ± 0.04 in the SHS exposed group (Figure 2B). These results were in concert with the functional myograph studies.

The mRNA level for the ET_A _receptor relative to GAPDH was significantly elevated after SHS in cerebral arteries (Figure 2A). The level of ET_A _receptor protein was 0.12 ± 0.02 relative to β-actin in the fresh air group and enhanced to 0.79 ± 0.02 after SHS (Figure 2B; *P *< 0.01). Taken together, the results demonstrate that SHS induces ET_A _receptor upregulation.

### MAPK signal pathway studies

**Figure 3:**
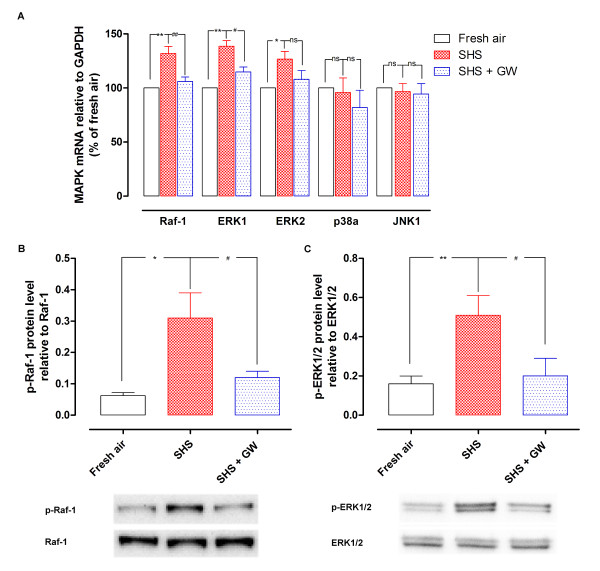
**The level of MAPK molecule (ERK1, ERK2, p38α and JNK1) mRNA expression in rat cerebral arteries **(A). Activation of Raf-1 protein expression in rat cerebral arteries (B). Activation of ERK1/2 protein expression in rat cerebral arteries (C). The cerebral arteries were isolated from groups of fresh air, SHS and SHS treatment with GW5074. The key MAP kinases mRNA was relative to GAPDH (*n *= 5-6); and the p-Raf-1 and p-ERK1/2 protein were relative to Raf-1 or ERK1/2 level (*n *= 5). *P < 0.05, **P < 0.01 versus fresh air group, ^#^*P *< 0.05 versus SHS group, ns not significant; SHS, secondhand smoke exposure; GW, GW5074 (0.5 mg/kg).

To investigate the underlying intracellular signal transduction mechanisms associated with the SHS-induced increase in ET_A _receptor expression, we first examined the mRNA levels of several key protein kinases such as Raf-1, ERK1, ERK2, p38α and JNK1 by real-time PCR. The results showed that SHS increased mRNA levels of Raf-1, ERK1 and ERK2 as compared to the fresh air group. SHS had no effect on p38α and JNK1 mRNA levels (Figure 3A).

In addition, we examined the phosphorylated (p)-Raf-1, p-ERK1/2, p-p38 and p-JNK, and their total protein expressions by Western blot in rat cerebral vessels. Total Raf-1 and ERK1/2 protein were unaltered in the SHS group compared to fresh air group. The phosphorylated protein of Raf-1 was enhanced to 0.31 ± 0.08 (SHS) from 0.06 ± 0.01 (Fresh air; *P *< 0.05, Figure 3B). The level of p-ERK1/2 protein relative to ERK1/2 in fresh air and SHS animals were 0.16 ± 0.04 and 0.51 ± 0.10 (*P *< 0.01), respectively (Figure 3C). In contrast, the protein level of p-p38 was 0.14 ± 0.02 (fresh air) and 0.16 ± 0.03 (SHS); the level of p-JNK protein was 0.21 ± 0.04 (fresh air) and 0.19 ± 0.03 (SHS). We did not find any significant difference in protein expression of p-p38 and p-JNK between fresh air and SHS groups. The results show that SHS induces enhanced expression of cerebrovascular ET_A _receptors *via *the Raf/ERK1/2 activation, but does not appear to involve JNK or p38 pathways in the present experimental conditions.

### Effect of GW5074 on SHS-induced effects

In order to further understand the role of Raf/ERK/MAPK signal pathway, we studied the inhibitory effect of daily GW5074 administration on SHS-induced responses. The results showed that the Raf-1 and ERK1 mRNA levels in cerebral arteries were significantly lower after GW5074 treatment in SHS exposed animals (Figure 3A). ERK2 mRNA showed a somewhat lower level after inhibition which was not different from the SHS group (*P *> 0.05). The mRNA levels of other protein kinases (p38α and JNK1) remained unaltered by GW5074 treatment in SHS. Western blotting confirmed the mRNA results. There was a significant decrease in the p-Raf-1 and p-ERK1/2 protein level after GW5074 treatment as compared with that of SHS (Figure 3B-C). The p-p38 and p-JNK proteins remained unchanged between the smoke exposure and treatment groups. The protein level of p-p38 was 0.16 ± 0.03 (SHS) and 0.18 ± 0.04 (SHS+GW); the level of p-JNK protein was 0.19 ± 0.03 (SHS) and 0.23 ± 0.05 (SHS+GW), respectively.

SHS or SHS with the Raf-1 inhibitor did not modify the weak S6c-induced contraction after 8 weeks of SHS combined with daily administration of GW5074 (Figure 1A). In contrast, treatment with GW5074 markedly attenuated SHS-induced enhanced cerebral vasoconstriction elicited by ET-1 at ET_A _receptors (Figure 1B). The E_max _of the ET-1 induced concentration-contractile curve in GW5074 treatment group was now the same as that seen in rats exposed to fresh air for 8 weeks (Table 2).

The mRNA and protein levels of ET receptors were also determined after treatment with Raf-1 inhibitor. The ET_B _receptor mRNA and protein levels were unchanged in the inhibitor group as compared to SHS or fresh air groups (Figure 2A-B). The mRNA level of ET_A _receptors was significantly reduced after inhibition of Raf/ERK/MAPK in the SHS group (Figure 2A). The protein level of ET_A _receptor was lower in the GW5074 treatment group but did not reach statistical significance (Figure 2B).

## Discussion

This is the first clear-cut demonstration that SHS increases the level of contractile ET_A _receptors in cerebral arteries *via *activation of the Raf/ERK/MAPK pathway. It is known that smokers or SHS exposure subjects have an increased risk to fall ill in stroke. However, the mechanisms behind this are poorly understood. Here, we show that the upregulation of ET_A _receptors with increased receptor-mediated vasoconstriction in the cerebral arteries observed after SHS exposure may be involved in SHS-related stroke. Specific inhibition of the Raf/ERK/MAPK pathway abolished the upregulation of ET_A _receptors in cerebral arteries of SHS exposed rats, while the other main MAPKs p38 and JNK were not affected.

Accumulating evidences indicate that both active and passive cigarette smoking are strongly associated with the origin and the development of stroke [1,13]. There is a clear relation between smoking-related stroke risk, the dose-response relationship existence, and the costs of the smoke exposure on individuals and society [24]. The present study was designed to imitate the manner of SHS exposure in man. It was found that animals required to be exposed to SHS for 8 weeks (200 min/day of passive smoke from the lit cigarette) to show ET_A _receptor changes. Two or 4 weeks of SHS did not alter ET receptor-mediated vasoconstriction in cerebral arteries. After 8 weeks of SHS exposure there was a significant increase in cerebral artery contraction mediated by ET_A _receptors. Basically, enhanced cerebral vasoconstriction mediated by receptors can be attributed to upregulated (presence of more) receptors and/or increased sensitivity of cerebral vessels in response to receptor agonist [22,25]. Since the contractile response mediated by receptors is considered a reflection of receptor expression in cerebral arteries, the receptor-mediated vasoconstriction is in accord with enhanced receptor levels. In agreement, results of mRNA and protein expressions of ET_A _receptors were in support of our hypothesis of more receptors. These results reveal that SHS upregulates the ET_A _receptor *via *a transcriptional mechanism. SHS exposure did not alter ET_B _receptor expression or the receptor-mediated contraction. This implies the method to culture cerebral arteries with tobacco extracts *in vitro *[14] differs from passive smoke exposure in the whole animal *in vivo*. Furthermore, SHS did not alter the K^+ ^induced contraction in any group which further suggests specificity in the receptor upregulation process.

It is known that the ET-1 levels in blood and CSF are increased in stroke; this may be further translated to an enhanced receptor-mediated contraction in cerebral arteries [26]. Transcriptional upregulation of ET_A _and ET_B _receptors has been reported in rat cerebral arteries after using some injury models like experimental cerebral ischemia and organ culture [10,19,27,28]. In all cases, the receptor upregulation occurred in the smooth muscle cells. The similar findings were confirmed in cerebral vessels from ischemic stroke patients [29,30]. Consequently, we believe that the ET_A _receptor was also increased in smooth muscle cells in the present study. Currently it reveal that SHS induces enhanced expression of ET_A _receptor mRNA and protein in cerebral arteries; this implies an important role in SHS-associated stroke. The significance remains to be tested in SHS-exposed animals using experimental stroke models; possibly they may show larger infarcts after an experimental stroke.

MAPKs have an important role in cerebrovascular receptor plasticity [3]. Particularly for ERK1/2, it located downstream of a dynamic chain of the kinases and is considered mainly mitogenic and has a predominant role in growth factor receptor signaling. We have demonstrated activation of ERK1/2 in cerebral arteries after MCAO and cerebral ischemia [18,21]. On this basis, the involvement of ERK1/2 pathway was assessed in the contractile receptor upregulation in artery culture [27]. Recently, a number of MAPK inhibitors were used to compare their ability to prevent the upregulation of various cerebrovascular vasoconstrictor receptors during organ culture [14,31]. In the present study we demonstrated SHS exposure induced ERK1/2 signaling activation by enhanced ERK1/2 phosphorylation. Moreover, we showed that SHS upregulated ET_A _receptors in rat cerebral arteries. It means SHS-induced ET_A _upregulation occurs through ERK1/2 activation. Meanwhile, we used a Raf-1 inhibitor GW5074 and confirmed that it is Raf/ERK1/2 signaling involved in the SHS-induced receptor changes, but not JNK or p38 pathway. This hypothesis is also supported by our recent *in vitro *discovery in cerebral arteries exposed to lipid-soluble smoke particles [28].

Raf-1 is associated ubiquitously in the Raf/MEK/ERK pathway. Raf phosphorylates MEK1/2, which in turn phosphorylates and activates ERK1/2 and then leads to activation of transcription factors [32]. The ERK1/2 pathway is a major effector of Raf. Transient activation of Raf-1 contributes to alterations in smooth muscle cell function, such as enhanced contraction and proliferation, whereas sustained activation results in differentiation *via *the regulation of various ERK substances [33]. We chose the Raf-1 inhibitor GW5074 to further demonstrate the involvement of ERK in the ET receptor upregulation after SHS. The specificity and efficacy of GW5074 for inhibiting Raf-1 *in-vivo *has been established in previous studies [32,33]. Lakey *et al. *and Chin *et al. *reported that GW5074 is a potent Raf-1 inhibitor and examined the effect of GW5074 on purified Raf-1 and confirmed that GW5074 selectively inhibits Raf-1 *in vivo*. In the present study, GW5074 attenuated the SHS-induced elevated cerebral artery contraction as well as increased mRNA mediated by ET_A _receptors. This strongly supports that SHS induces ET_A _receptor upregulation *via *the Raf/ERK/MAPK pathway. We demonstrated the mRNA of Raf-1 and ERK1/2 was increased after SHS, but the total Raf-1 or ERK1/2 proteins were not changed. We think the former measurement reflects steady state and thus that may also be other changes such as in degradation or mRNA stability.

The enhanced phosphorylation of Raf-1 and ERK1/2 suggests the Raf/ERK1/2 pathway has been activated. The kinases elicit some of their effects through phosphorylation of transcriptional regulation. Currently, Raf-1 inhibitor GW5074 reduced phosphorylation of ERK1/2 as well as Raf-1. The GW5074-induced declined phosphorylation of ERK1/2 should be attributed to the upstream inhibition of ERK1/2. However, the reason to explain the reduced Raf-1 phosphorylation is not sure. We think it may be some upstream influences or feedback mechanisms when blocking Raf-1 activity by GW5074. It could be a partial reason for decreased Raf-1 phosphorylation. Moreover, we performed *in vivo *treatment in the animals. It may also be some possible indirect effects of GW5074 that altered Raf-1 phosphorylation when administrated with the inhibitor *in vivo*. However, the overall data agree with the involvement of Raf/ERK/MAPK in SHS.

## Conclusions

The present study is the first to show that passive smoke exposure upregulates ET_A_, but not ET_B _receptors, in rat cerebral arteries. The upregulation of ET_A _receptors occurs *via *activation of the Raf/ERK/MAPK pathway. This mechanism may provide new options for treatment of SHS-associated cerebrovascular diseases.

## Methods

### Animals

Male Sprague-Dawley rats (8-weeks old) were provided by the Animal Center of Xi'an Jiaotong University College of Medicine (China). All animal procedures were approved by the Animal Ethics Committee of Xi'an Jiaotong University.

### Passive cigarette smoke exposure model

Animals were exposed for 2 weeks, 4 weeks or 8 weeks to SHS or fresh air (control). In a preliminary study, we did not find significant difference of cerebral contractility mediated by ET receptors in the 2 or 4 weeks groups. Therefore, these data are only mentioned briefly below. In the subsequent study, 30 rats were randomly divided into 3 groups of 10 rats in each group are exposed for 8 weeks: (i) fresh air exposure injected with saline vehicle; (ii) smoke exposure injected with saline; (iii) smoke exposure and treatment with GW5074 (0.5 mg/kg, i.p.). The rats were exposed to cigarette smoke from commercially available filter cigarettes (Marlboro, 1.0 mg of nicotine and 12 mg of tar content) in a plastic chamber (115 × 50 × 65 cm; 0.37 m^3^). Each cigarette was freely burning for 15 min, then the cigarette smoke was allowed to diffuse in the whole chamber for another 25 min. Fresh air was then present for 20 min after every SHS exposure [34]. For each smoke exposure, 2 cigarettes were lit simultaneously. The rats were repeatedly exposed to the smoke 5 times every day for up to 8 weeks. The total SHS exposure was therefore 200 min/day. The rest of time animals were exposed to fresh air. In the fresh air group, rats were exposed only to room air. For the treatment group, GW5074 was administrated to the animals once every day for 8 weeks in addition to the same condition of SHS exposure. The dosage of GW5074 was based on a previous study [32,34]. This exposure type may resemble SHS exposure and that the level of nicotine in the animals compare well with at seen in plasma of human smokers.

### Harvest of cerebral arteries

After the exposure period (fresh air, SHS and SHS treated with GW5074), rats were anesthetized with CO_2_, sacrificed in a cage filled with dry ice and then decapitated. The basilar arteries, middle cerebral arteries and circle of Willis arteries were dissected free from the brain and chilled in ice-cold bicarbonate buffer solution [11]. Some of the basilar arteries were cut into cylindrical segments for *in vitro *pharmacology studies. The remaining part of the basilar arteries, the middle cerebral arteries and the circle of Willis arteries were snap frozen at -80°C for real-time PCR and Western blot examinations.

### Cerebral artery contractile function studies

The myograph experiments were performed at the Department of Pharmacology, Jiaotong University, while the other experiments were done in the Lund University. Wire myograph (Danish Myo Technology A/S, Aarhus, Denmark) was a sensitive system for recording the vessels contractile properties. The cerebral artery segments were mounted on two thin wires in temperature controlled (37°C) myograph baths containing 5 mL bicarbonate buffer solution. Detailed method has been described before [10,35]. The viability of arterial segments was then tested by exposure to a potassium-rich (63.5 mM K^+^) buffer solution.

Concentration-response curves were obtained by the cumulative administration of the selective ET_B _receptor agonist S6c, and the combined ET_A _and ET_B _receptor agonist ET-1. To study ET_A _receptor-mediated contraction, the experiment started with the desensitization of the ET_B _receptors by performing a concentration-response curve to S6c firstly [36]. The ET_A _receptor and the ET_B _receptor antagonists (FR139317 and IRL2500) were used to show receptor specificity [22,23].

### Real-time PCR

Total RNA was extracted from cerebral vessels using RNeasy Mini kit, following the supplier's instruction (Qiagen, Valencia, USA). Details were described before [10]. Reverse transcription of total RNA to cDNA was performed using the TaqMan Reverse Transcription Reagents (Applied Biosystems, Branchburg, USA) in a Perkin Elmer 2400 GeneAmp PCR system (Perkin Elmer, MA, USA). Real-time PCR was performed using the GeneAmp SYBR^® ^Green kit in a GeneAmp 7300 Sequence Detection System Specific primers for the ET_A _and ET_B _receptors were designed as follows: ET_A _receptor, forward: 5'-GTC GAG AGG TGG CAA AGA CC-3', ET_A _receptor, reverse: 5'-ACA GGG CGA AGA TGA CAA CC-3', ET_B _receptor, forward: 5'-GAT ACG ACA ACT TCC GCT CCA-3', ET_B _receptor, reverse: 5'-GTC CAC GAT GAG GAC AAT GAG-3'. Primers of ERK1, ERK2, JNK1 and p38α protein kinases genes were purchased from RT^2 ^qPCR Primer Assay. As an endogenous standard, the primers of glyceraldehyde-3-phosphate dehydrogenase (GAPDH) were designed as follows: GAPDH, forward: 5'-GGC CTT CCG TGT TCC TAC C-3', GAPDH, reverse: 5'-CGG CAT GTC AGA TCC ACA AC-3'. The mRNA content was calculated relative to the amount of GAPDH. These have been analyzed previously and verified using 3 different standards [10].

### Western blot

The protein of cerebral vessels was extracted as described before [37]. After gel eletrophoresis and protein transfer, membrane was then blocked in 5% non-fat milk. Subsequently, the membrane was incubated at 4°C overnight with primary antibodies: rabbit anti-ET_A_, rabbit anti-ET_B_, rabbit anti-p-Raf-1, anti-p-ERK1/2, rabbit anti-p-JNK1/2/3, rabbit anti-p-p38, mouse anti-β-actin, rabbit anti-Raf-1 or mouse anti-ERK1/2. Then, membranes were incubated with horseradish peroxidase-conjugated anti-rabbit or anti-mouse secondary antibodies. Finally, membranes were developed and visualized using a Fujifilm LAS-1000 Luminescent Image Analyzer (Fujifilm, Stamford, USA) and the band intensity was quantified by Image Gauge Version 4.0.

### Statistical analysis

All data are expressed as means ± SEM and *n *refers to the number of rats. Unpaired Student's *t*-test was applied to compare two sets of data. One-way analysis of variance (ANOVA) with Dunnett's post-test was used for comparison of more than two data sets. Two-way ANOVA with Bonferroni's post-test was used to compare the two corresponding data points at each concentration of the two curves. *P *< 0.05 was considered as statistical significance.

## Authors' contributions

LC carried out the experiments and data analysis, participated in design of the study and drafted the manuscript. CBX supervised the whole work, conceived and designed the study. YZ helped with revising the manuscript. YXC took part in designing the study, helped with performing the myogragh researches and draft the manuscript. LE provided intellectual input in the study and helped with revising the manuscript. All authors contributed in writing the manuscript and approved the final version.
